# A Rapid Public Health Needs Assessment Framework for after Major Earthquakes Using High-Resolution Satellite Imagery

**DOI:** 10.3390/ijerph15061111

**Published:** 2018-05-30

**Authors:** Jian Zhao, Fan Ding, Zhe Wang, Jinghuan Ren, Jing Zhao, Yeping Wang, Xuefeng Tang, Yong Wang, Jianyi Yao, Qun Li

**Affiliations:** 1 Chinese Center for Disease Control and Prevention, Beijing 102206, China; zhaojian@chinacdc.cn (J.Z.); dingfan@chinacdc.cn (F.D.); wangzhe@chinacdc.cn (Z.W.); renjh@chinacdc.cn (J.R.); zhaojing@chinacdc.cn (J.Z.); wangyp@chinacdc.cn (Y.W.); 2 Health and Family Planning Commission of Sichuan Province, Chengdu 610041, China; sccdctxf@163.com; 3 Institute of Geographic Sciences and Natural Resources Research, CAS, Beijing 100101, China; wangy@igsnrr.ac.cn

**Keywords:** earthquake, public health emergency management, high-resolution remote sensing, evaluation

## Abstract

*Background*: Earthquakes causing significant damage have occurred frequently in China, producing enormous health losses, damage to the environment and public health issues. Timely public health response is crucial to reduce mortality and morbidity and promote overall effectiveness of rescue efforts after a major earthquake. *Methods*: A rapid assessment framework was established based on GIS technology and high-resolution remote sensing images. A two-step casualties and injures estimation method was developed to evaluate health loss with great rapidity. Historical data and health resources information was reviewed to evaluate the damage condition of medical resources and public health issues. *Results*: The casualties and injures are estimated within a few hours after an earthquake. For the Wenchuan earthquake, which killed about 96,000 people and injured about 288,000, the estimation accuracy is about 77%. 242/294 (82.3%) of the medical existing institutions were severely damaged. About 40,000 tons of safe drinking water was needed every day to ensure basic living needs. The risk of water-borne and foodborne disease, respiratory and close contact transmission disease is high. For natural foci diseases, the high-risk area of schistosomiasis was mapped in Lushan County as an example. Finally, temporary settlements for victims of earthquake were mapped. *Conclusions*: High resolution Earth observation technology can provide a scientific basis for public health emergency management in the major disasters field, which will be of great significance in helping policy makers effectively improve health service ability and public health emergency management in prevention and control of infectious diseases and risk assessment.

## 1. Introduction

Earthquakes are among the most terrifying and destructive of all natural hazard-caused disasters, which can arguably lead to incalculable environmental damage, construction damage, loss of life, population displacement, overcrowding, propitious circumstances for an epidemic and threats to health [1]. China, one of the world’s most earthquake-prone countries [2], has experienced significant such damage events in recent years, such as the 2008 Wenchuan earthquake [3], the 2010 Yushu earthquake [4], and the 2013 Ya’an earthquake [5]. The Wenchuan earthquake in the Sichuan province of China affected the most people [1].

Emergency management activities relating to earthquake include preparedness, mitigation, response and recovery, e.g., the FEMA approach in USA, EU Civil protection in Europe, and Emergency Management in Australia [6]. After a major earthquake, from the viewpoint of public health, timely emergency response is crucial to reduce casualties and guide relief efforts [7]. A proper multistage continuous assessment [1] to identify urgent public health issues is the most critical component of earthquake response.

In the early hours after an earthquake, collecting information and evaluating the extent of damage is essential for anticipating the healthcare needs of survivors, managing critical conditions [8], and allocating limited resources. Casualties and injuries estimation is one of the most crucial processes to measure the severity of damage [9]. The methods used to determine the loss of life caused by earthquakes can be divided into two categories. In the first category, the empirical function is proposed based on the relationship between earthquake parameters and the number of casualties reported by historical data; In the second category, the research focuses on the relationship between building damage and the number of casualties [10], which is more accurate and reliable as building damage is main cause of earthquake casualties and injuries in China [11,12].

Earthquakes severely destroy or overwhelm the health infrastructure and medical service system, which hampers disaster victims’ timely access to health services. Injury and soft tissue infections are expected during the first few days after earthquake. In contrast, water source and foodborne diseases, respiratory and close contact diseases, insect-borne and natural focus diseases are anticipated for up to one month after an earthquake [13,14]. Identifying areas where medical services are deficient [15,16] is important to ensure the most appropriate medical rescuers quickly reach the disaster area where they are most needed.

The public health issues after an earthquake usually include the interruption of access to safe water and sanitation facilities, increased exposure and susceptibility to disease, population displacement with overcrowding, etc. Landslides triggered by an earthquake can cause the displacement of water pipes and building damage, resulting in water supply difficulties [17]. The damage to drinking water supply systems and sanitation facilities on a large scale can hinder disaster victims from accessing safe water and food, which will increase the risk of waterborne and foodborne disease outbreaks. Chaos caused by population displacement and overcrowding [18] is also associated primarily with communicable disease outbreaks. The first response in preventing an outbreak is to provide adequate shelter as soon as possible to the affected population. Emergency facility locations for earthquake victims need to be well planned [19].

Valid and rapid information acquirement is critical to enable decision-making and resource prioritization by health care providers and emergency management officials during the response immediately after a disaster [20]. Geographic information systems (GIS) provide a useful tool to help with this issue [21]. Gridded population distribution maps can be used in public health applications and risk evaluation [22,23,24] to predict the affected population. Remote sensing images are an important information source for an accurate overview of earthquake-induced damages [25] using optical, LIDAR, or synthetic aperture radar (SAR) from satellite or aerial platforms [26]. In recent years, with the development of a high resolution Earth observation system in China, high-resolution satellite imagery has been used in disaster emergency monitoring and evaluation [27], which improves the textural and spatial feature extraction to help identify damaged regions more accurately [28,29].

This paper aimed to build a framework for rapid public health needs assessment, which objectives include: (1) estimating casualties and injuries; (2) identifying damaged medical facilities; (3) estimating drinking water needs; (4) identifying areas at risk of disease; and (5) identifying temporary settlement sites. This assessment will provide technical support for public health emergencies management after earthquakes.

## 2. Framework of Rapid Public Health Needs Assessment

**Figure 1:**
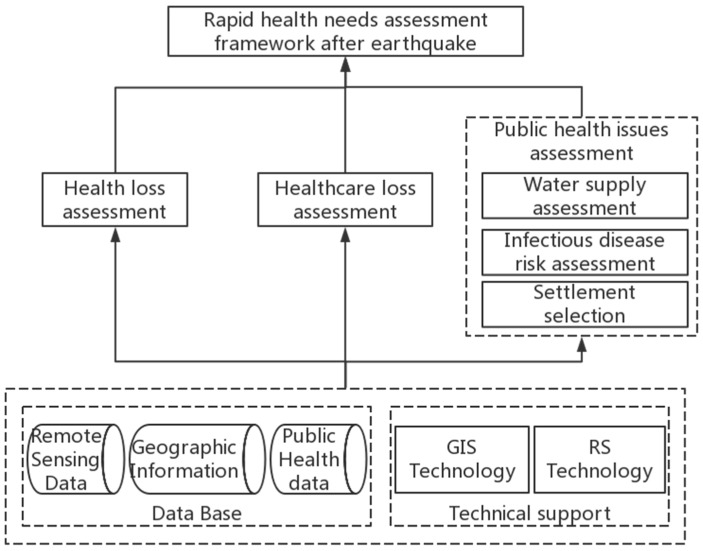
Framework of rapid public health needs assessment after major earthquake.

In order to evaluate public health needs rapidly after an earthquake, an assessment framework, shown in Figure 1, was established based on GIS technology and high-resolution remote sensing images. Health losses, including casualties and injuries, were estimated with great rapidity. Health care loss aims to identify areas where medical services are deficient. Then, the public health issues, including water supply, infectious disease risk and settlement selection, were evaluated to improve the management of rescue efforts, and organization of subsequent restoration activities.

## 3. Materials and Methods

### 3.1. Study Area

**Figure 2:**
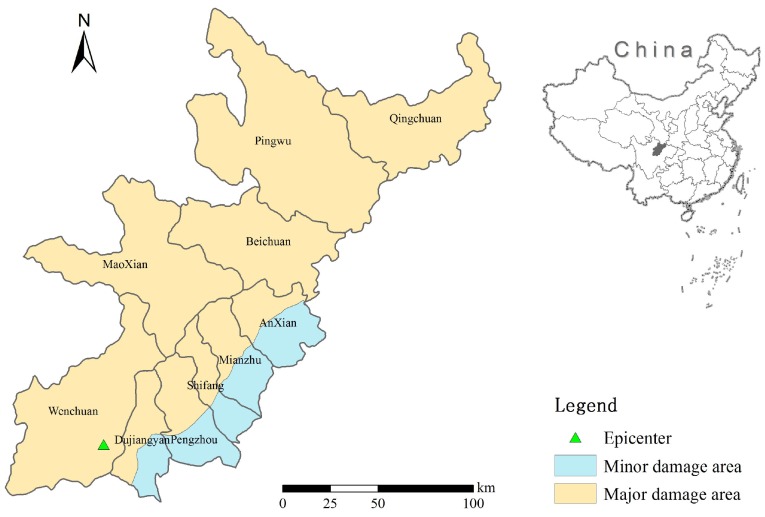
Study area with major and minor damage area divisions.

The magnitude 7.9 Wenchuan earthquake which took place at 1428 h Chinese Standard Time on Monday, 12 May 2008, in the Sichuan Province of China (Epicenter 31.0° N, 103.4° E) was the most destructive in China since 1976 [3,30]. According to the national earthquake relief headquarters, 137 days after the earthquake, 69,227 people were confirmed to have been killed, 374,643 people were injured, 17,923 people were missing, 46,240,000 people were affected, and 15,100,000 people had to leave their homes to seek safer shelter [31]. This event caused huge damage and loss of life, and a massive relief and recovery operation was mounted by the Chinese government [32]. The Sichuan Province was the hardest-hit area, with the greatest number of casualties in 10 counties, including Wenchuan, Beichuan, Qingchuan, Mianzhu, Shifang, Dujiangyan, Pingwu, Anxian, Pengzhou and Maoxian [33], shown in Figure 2.

The magnitude 7 Ya’an earthquake took place at 0802 h Chinese Standard Time on 20 April 2013 in the province of Sichuan, China. In this incident 196 people were killed, 11,470 people were injured, and more than 1,500,000 people were affected [5]. The epicenter (30.3° N, 103.0° E) was in Lushan County. Lushan and Tianquan counties are two endemic areas for schistosomiasis with high historic transmission levels, especially Lushan County where the *Oncomelania* snail distribution/ density was the highest in Sichuan Province [34].

### 3.2. Data Collection and Processing

#### 3.2.1. Satellite Data

We collected pairs of pre- and post-earthquake QuickBird images on 26 June 2005 and 3 June 2008 to detect the damaged area in Yingxiu County (Wenchuan). QuickBird is a high-resolution commercial satellite launched on 18 October 2001. It has been acquiring optical images of urban areas with maximum spatial resolution of 0.6 m, which can be used to detect building damage after earthquakes. Aerial images on 20 April 2013 were acquired from the Chinese Academy of Sciences Academy of OPTO-Electronics (Beijing, China) for Longmen township, affected by the Ya’an earthquake. 

#### 3.2.2. Infectious Disease Incidence Data

Infectious disease incidence data were derived from the national web-based infectious disease surveillance network maintained by the Chinese Center for Disease Control and Prevention (China CDC, Beijing, China). Data used in this article included infectious diseases report monthly statistics in Sichuan Province from 2004 to 2007.

#### 3.2.3. Population Data

Gridded population data in China on 2000 were provided by the Chinese Academy of Sciences Data Center for Resources and Environmental Sciences, (RESDC, Beijing, China, http://www.resdc.cn). The gridded population data has a spatial resolution of 1 km, which was transformed from census data based on the relationship between demographical data and land use types [35]. Demographic data on 2008 was collected from the National Bureau of Statistics of the People’s Republic of China (Beijing, China).

#### 3.2.4. Geographic Information Data

The availability of appropriate and accurate topographic elevation data on the affected area is of uttermost importance for image analysis and mapping tasks. The terrain group variables, digital elevation models of the terrain and slopes were obtained from Shuttle Radar Topography Mission (SRTM) digital elevation model (DEM) [36]. The elevation data with resolution of ~90 m is of very high value for image processing and map generation. The DEM variable is given in meters and the slope variable is given in degrees. Administrative division data used the 1:1,000,000 administrative zone map.

### 3.3. Estimating Earthquake Casualties and Injures

In order to improve the efficiency of our estimations, we developed a two-step casualty estimation method. In the first step, we combined the geomorphologic features of the earthquake zone, seismic parameters and gridded population data [37,38] into Building Damage Evaluation Model [39] and Casualty Estimation Model [40] to predict casualties in the disaster area very quickly. In the second step, the high-resolution remote sensing data acquired pre and post-earthquake was used to extract damage building information. The number of injuries caused by earthquake generally tripled from casualties according to experience.

The Casualty Estimation Model in a 1 km grid is given as:

$$\log\left(\mathrm{RD}\right)=9.0{\left(\mathrm{RB}\right)}^{0.1}-10.07$$

$$\mathrm{ND}=f_{t}f_{p}\left(\mathrm{RD}\right)P$$

where ND is the casualties, RD is the earthquake mortality, RB is the rate of building collapse, P is the gridded population, $f_{t}$ is the correction factor for earthquake occurrence time, $f_{p}$ is the correction factor for population density.

The Building Damage Evaluation Model is given as:

RB=RB[S|I]

where, RB[S|I] is the seismic damage matrix when seismic Modified Mercalli Intensity (MMI) is I and the building class is *S*.

However, in the actual seismic damage evaluation, investigation and statistics of national housing construction are not accurate due to cost, time and other reasons. Here, we divided the housing construction into four types:

(1) Type A are a multi-storey reinforced concrete houses that have steel and reinforced concrete structures, such as high-rise steel and reinforced concrete frame shear wall structures, reinforced concrete shear wall structures, high-rise and multi-storey reinforced concrete frame structures, etc. This kind of structure has the best seismic performance of all structures;
(2) Type B are multi-storey masonry buildings. They include brick structures, industrial buildings, public buildings, etc. This kind of structure is the most abundant in cities and their seismic performance is inferior to that of A class buildings.
(3) Type C are single homes; these structures mainly include the lime mortar masonry brick buildings, the 24 cm thick brick structures of empty houses and classrooms, hollow brick wall structures, etc.
(4) Type D are buildings with adobe, earth-rock structure. These includes the raw soil structures often found in the countryside, such as adobe, adobe caves, rock structures. The seismic performance of these is the worst type of all types of structure.

Earthquakes can cause dramatically different casualty rates, depending on their location. In this study, we defined the plains with the slope less than 5° and the height less than 700 m as the minor damage area; the rest of the mountain area was defined as the major damage area. In the minor damage area, housing construction is mainly of type C; in the major damage area, housing construction is mainly of type D. Type C and D structural earthquake damage matrices are presented in Table 1.

For the same MMI, geographical conditions and structure type, the higher the population density is, the greater is the number of casualties. Therefore, the influence of population density factors should be considered in the evaluation. The correction factor for population density $f_{p}$ is presented in Table 2.

Earthquake casualties are related to the indoors population at the time of the event, which is determined by people’s activities, so the correction factor is 1 in daytime, and the nighttime correction coefficient under different MMI scenarios are shown in Table 3.

In the second step, the pre- and post-earthquake QuickBird images on 26 June 2005 and 3 June 2008 were used to extract damaged buildings. The high-resolution remote sensing data was preprocessed, then collapsed buildings were interpreted using an object oriented extraction [41] algorithm. First, the high-resolution images were classified with multi-scale segmentation technology; then a CART decision tree was used to establish a rule set according to the features and threshold selection; a fuzzy function algorithm was applied to classify the images after segmentation, thus to extract buildings.

### 3.4. Rapid Medical Resource Damage Assessment

In the rapid medical resource damage assessment after earthquake, historical data and health resources reports were reviewed, including distribution of medical institutions and number of beds. In order to evaluate the damage condition of medical resources, the minor damage area and major damage area was defined previously. When the seismic category is larger than magnitude 7.0, the medical resources in a major damage area cannot work properly, while in the minor damage area the damage rate was defined as 30 percent here.

### 3.5. Public Health Needs Assessment After an Earthquake

Public health needs were evaluated on the basis of earthquake injures and medical resource damage assessment, including water supply situation, outbreaks of infectious diseases and rescue layout.

#### 3.5.1. Water Supply Assessment

A functioning water supply system is essential for health protection in cases of violent earthquakes. A temporary water source will be needed until the water supply system resumes normal activity as the water sources in a mountainous area could be completely destroyed. The temporary water source setting selection must consider the distribution of water, housing collapses in the affected areas and the risk of secondary hazards. A source should be near a river (200 m), near a road (200 m) and close to the settlement (within 300 m) in order to access safe water conveniently. The minimum size of temporary water source installations should be more than 50 m^2^. What’s more, the settings should be located in the upper reaches of settlements to ensure the water quality and 200 m away from landslide areas to keep them safe.

Drinking water demand was evaluated based on the minor damage area and major damage area. In the major damage area, water systems would be seriously damaged and there would be a lack of a centralized water supply which is difficult to provide in the short term, so the water supply mainly depends on bottled water. In this extreme situation, supplying a survival level of safe drinking water is of critical importance when there may not be sufficient water available to meet basic needs. According to the minimum health need standards, average water use for drinking, cooking and personal hygiene in any household is at least 15 L per person per day [42]. In the minor damage area, the water supply system could recover in a short time; average water use is calculated as 7 L per person per day. This assessment does not consider the additional safe drinking water demand for rescue workers.

#### 3.5.2. Risk Assessment of Infectious Disease

The historical situation of communicable diseases was overviewed in Sichuan Province and the severely affected areas. The infectious disease incidence data in the past 10 years before the disaster was collected. Semi-structured interviews and discussions among subject matter experts based on surveillance data were conducted to assess the risk of disease outbreak.

For natural foci diseases, such as plague and schistosomiasis, the destruction of the habitat may cause the transmission or epidemics. Lushan County with the highest *Oncomelania* snail distributions/densities in Sichuan Province and was affected in the Ya’an earthquake. Literatures were reviewed to map the historic distribution of *Oncomelania*, the water distribution and the residential distribution. Pairs of pre- and post-earthquake aerial remote sensing data images were acquired and interpreted to detect the range of infected water. High-risk area of schistosomiasis was mapped within 500 m of river and within 500 m of residential area.

#### 3.5.3. Settlement Selection after an Earthquake

Here, we considered the temporary settlements selection with regional medical rescue ability. This selection follows several rules: (1) *Nearest principle*. The settlement should be close to the disaster area, so the affected population should be easily transported to settlements; (2) *Safety Principle*. High MMI earthquakes can result in a large number of casualties and infrastructure damage when they occur in close proximity to the epicenter [9]. There is usually a long period of aftershocks and a lot of secondary hazards. Thus, in the post-disaster resettlement plan, security of settlements must be considered a priorty. Seismic zones, landslides and debris flows areas should be avoided, such as flood areas of reservoirs and barrier lakes, major pollution sources, high pressure corridors, high-pressure gas pipelines, etc. and they should be preferably near the main highway; (3) *Convenient transportation*. Transitional resettlement sites shall be elected in areas where traffic conditions are convenient and the people affected by the disaster can be restored to production and life; (4) *Size limitations*. The size of the site should not exceed the environmental bearing capacity of the site, and the destruction of ecological resources should be minimized so as not to cause irreversible effects. One should try to occupy not farmland, avoid nature reserves, drinking water source area protection area and ecologically fragile areas. The guaranteed minimum per capita area should consider the residents’ birth and life requirements, as well as the need for social interaction and psychological recovery [43,44].

We proposed a rescue site selection indicator system to select the possible layout area of the rescue point based on a spatial analysis method, as shown in Table 4.

(1) It should be within 50–500 m of the damaged residential areas to facilitate the transport of the wounded;
(2) In order to ensure the smooth access of medical supplies and relief workers to the rescue point, a road traffic area should be selected and be within 200 m of the highway.
(3) To prevent the secondary hazards such as landslides, debris flows, lake threats to personnel, the selected area should be 100 m away from a river system, and any landslide area should be 200 m away from the secondary disaster risk area;
(4) To avoid high slopes, and the vegetation that is rich in mountains, the emergency rescue area that refers to the smallest size square hospital, the emergency rescue point location area shall be no less than 2500 m^2^.

## 4. Results

### 4.1. Earthquake Casualties and Injuries Estimation

In the emergency phase, the casualties and injures are estimated within a few hours after an earthquake. The elevation and slope information of the terrain data were extracted. The study area was divided into a major damage area and minor damage area, as shown in Figure 2. The major damage area in the northwest is about 26,980 km^2^, accounting for 73.79%. The minor damage area in the southeast is about 9581 km^2^, accounting for 26.21%.

Building collapse rate of 1 km grids was estimated based on the Building Damage Evaluation Model. The seismic MMI and regional geographic features were taken into consideration. In the mountain area near the epicenter, the collapse rate of buildings can reach 98.5%. The collapse rate of buildings in the plain far from the epicenter was low, about 50.71%.

**Figure 3:**
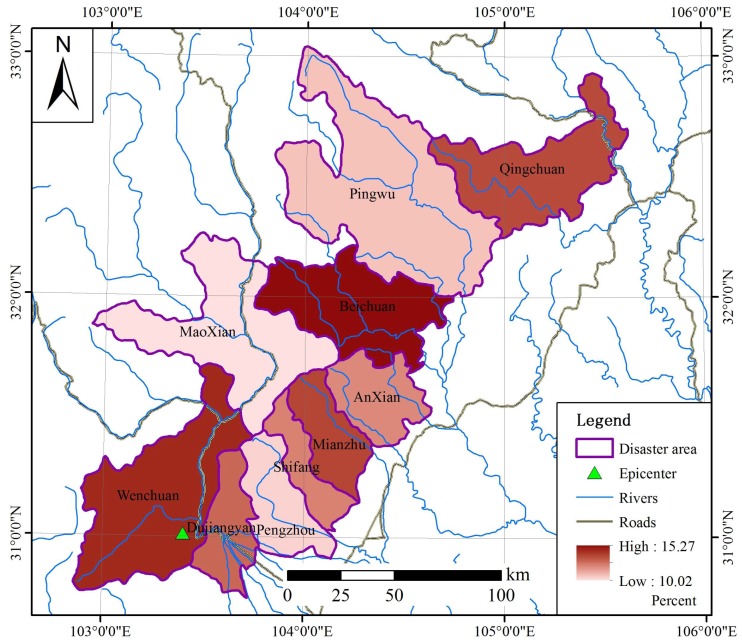
Mortality rate of assessment area in Wenchuan Earthquake.

The average housing collapse rate in counties was calculated. Wenchuan County, Beichuan County and Qingchuan County were the most severely damaged areas, with a collapse rate higher than 88%. Pengzhou County has a lowest collapse rate of 74.21%. The death rate and the injury rate in the study area were evaluated based on Casualty Estimation Model, as shown in Figure 3. According to our estimation, the Wenchuan earthquake killed about 96,000 people and about 288,000 people were injured, as shown in Table 5.

**Figure 4:**
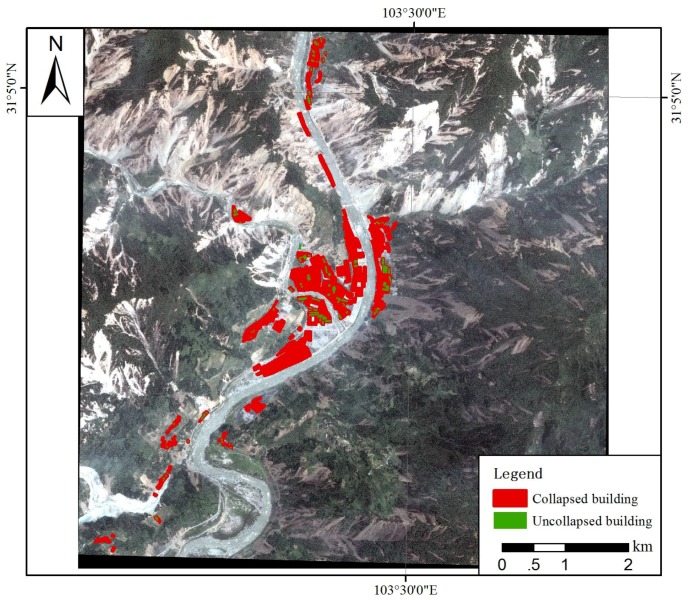
Results of house collapse interpretation in Yingxiu Town using high-resolution remote sensing images.

The interpreted collapsed buildings are shown in Figure 4. The infrastructure collapse rate in Yingxiu Town of Wenchuan County was 91.93%.

### 4.2. Rapid Medical Resource Damage Assessment

There were 294 medical institutions located in the study area, of which 242 (82.3%) were severely damaged, as shown in Table 5. The major damage area in the northwest is deep in the mountainous hinterland, and completely isolated from the outside traffic. The medical and sanitation equipment is almost all completely destroyed. In the southeastern part of the country, health resources were less damaged than in the northwest, and about 30% (114/166) of the medical and health facilities were damaged.

### 4.3. Public Health Needs Assessment after Earthquake

#### 4.3.1. Water Supply Assessment

**Figure 5:**
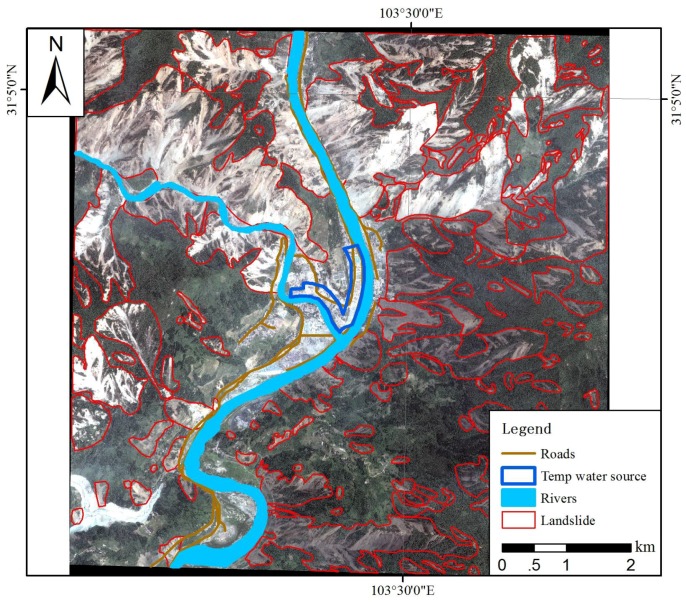
Temporary water source setting location.

For the major damage area, temporary water source setting was selected based on the indicators discussed previously, as shown in Figure 5. According to our estimation, in the 10 counties severely damaged by the Wenchuan earthquake, there were about 3,500,000 people that needed about 40,000 tons of safe drinking water (Table 5) every day to ensure their basic living needs. In addition to the self-provided wells and stored bottled water, the demand for drinking water needs to be solved. The assessment of water requirements is aimed at identifying the availability (quantity and quality) of water related to demand and providing decision support for the development of relief measures and public health services.

#### 4.3.2. Risk Assessment of Infectious Disease

Risk assessment results of infectious disease after the Wenchuan earthquake (Table 6) shows that the risk of water-borne and foodborne disease is high, with the greatest potential risks corresponding to bacillary dysentery and other types of infectious diarrhea. At the same time, the risk of cases increasing or even local outbreaks is high for cholera, hepatitis A, typhoid and paratyphoid. The risk of respiratory and close contact transmission diseases is high too, especially for acute respiratory infections, mumps, tuberculosis and acute hemorrhagic conjunctivitis. The risk of insect-borne disease and natural focal disease is considered low [45].

**Figure 6:**
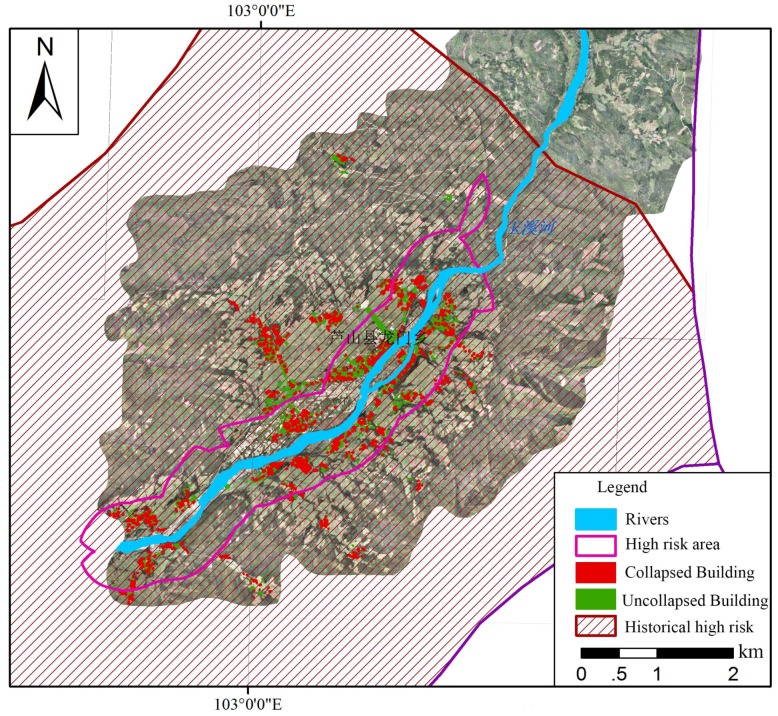
Risk assessment of natural foci diseases after the Ya’an earthquake.

For natural foci diseases, the destruction and change of the ecological environment caused by the earthquake may increase the outbreak and epidemic risk of infectious diseases. The historic distribution of *Oncomelania* and high-risk areas for schistosomiasis are mapped in Figure 6.

#### 4.3.3. Settlement Selection after an Earthquake

**Figure 7:**
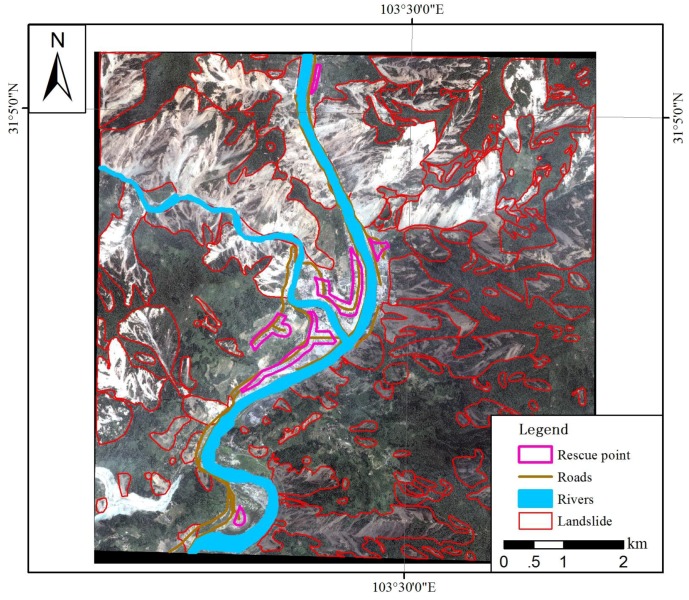
Distribution of the disaster relief point layout.

Passable roads, river systems and landslides were interpreted based on before-after earthquake satellite image pairs, then the rescue point selection indicators were calculated. According to the principles of settlement selection, the scope of the disaster relief point layout was determined, as shown in Figure 7.

## 5. Discussion

This paper discussed a framework of rapid public health needs assessment after earthquakes using GIS technology and high-resolution remote sensing images. In post-disaster situations, this assessment is essential in the rapid implementation of control measures through re-establishment and the improvement of primary healthcare delivery [46]. Remotely sensed data have been increasingly used for monitoring, surveillance, or risk mapping. Capabilities of geographic information systems extended its use into operational disease surveillance and control. This high-resolution image solution would guide the application of Chinese satellites in public health.

Casualties and injuries estimation after an earthquake is crucial to support the design of the public health emergency response and needs to be evaluated as soon as possible. The casualty estimation model depends on the building damage situation, because in China, buildings and infrastructure are mainly responsible for the loss of lives and injuries [47] and therefore important factors of vulnerability to earthquakes [12]. Population distribution and its variations are another factors that influence the casualty rate. Thus, the proposed two-step estimation method considering both building collapse and population distribution could be completed in several hours accurately. The number of injuries in the 10 districts and counties affected in the Wenchuan earthquake was estimated by this paper as 288,438, compared with the injury census of 374,643 [31], so the accuracy of our estimation is about 77%.

Damage to public infrastructure often leads to disruptions in medical care. In our evaluation, the collapse rate of medical buildings was 82.3%, while in the investigation it was 67.5% [1]. This serious event on such a scale needs extraordinary efforts to cope with it, often with outside help or international aid.

The earthquake damage of the water supply system causes great difficulty and influences the survival of the victims. In most cases, the main health problems are caused by poor hygiene due to insufficient water and by the consumption of contaminated water. The effect on water quality of an earthquake is mainly manifested in the following two aspects: on the one hand, the direct impact of the ecological balance breakdown causes a great change to the water environment; on the other hand, damage to the industrial infrastructure and mining enterprises can result in a large number of pollutants being released into the local environment which can have a serious influence [1]. Temporary water source setting assessment considered safety and convenience, which would further guide post-earthquake recovery and reconstruction. Actually, after the Wenchuan earthquake, the water sources were capable of meeting the demands of local residents [48], but the destruction of water systems limited the supply of drinking water. What’s more, in the extreme cases after the earthquake, victims did not trust water supply, and lacked knowledge of water purification treatment. Thus, basic water demands should be estimated according to the minimum health need standards. The assessment of water requirements is aimed to provide a reference for the storage and transportation of relief materials. In addition, more than 20,000 medical and health workers participated in the earthquake response, as well as emergency workers from other departments, such as electric power, water conservancy, traffic, and for these there was a lack of statistics. Thus, this assessment does not consider the demand of safe drinking water for rescue workers.

The primary driver to influence the risk for communicable diseases after natural hazard-caused disasters is the interplay of safe water and sanitation facilities, the degree of crowding, the underlying health status of the population, and the availability of healthcare services [49]. There could be outbreaks of infectious diseases after an earthquake, such as acute watery diarrhea [50], hepatitis E [51], and coccidiomycosis [52]. Epidemiological studies of disasters have led to important environmental and policy changes [53]. However, natural hazard-caused disasters do not import of diseases [54]. Thus, the prompt assessed the risk for different contagious diseases after the Wenchuan earthquake through historic surveillance data would be helpful for situation awareness. Delay in public health assessments will decrease the efficiency of disease prevention efforts [55]. The incidences of water-borne and foodborne disease, bacterial dysentery and other infectious diarrhea disease were higher than those of the unaffected area in the first four weeks. Factors contributing to disaster severity include environmental damage, seasonal variation and crowded conditions. No outbreak of infectious disease or other public health emergency was reported in the Wenchuan earthquake [56].

For natural foci diseases, schistosomiasis was selected to be evaluated. After the Ya’an earthquake, the damaged streams and ditches caused a widening of the water surface which would potentially leading to the spread of *Oncomelania* snails. Meanwhile, the people who were relocated due to the earthquake may have higher exposures to the contaminated environment. High risk areas were mapped to provide clues to guide disaster management.

For impacts on public health, injury and psychosocial damage are important effect [57], while an indirect threat to epidemics comes from possible disruption of food and water security and environmental safety. This threat is increased by the displacement of populations as a result of damage to residential buildings. In China, measures generally are in place to prevent this from happening [58]. The quality of earthquake response depends on the settlement selection strategy. Here, indicators were selected following several principles. Through the comparison of remote sensing images, we can see that the location of our site is consistent with the distribution of relief tents set up after the actual investigation.

This methodology is useful for ex post evaluations, some application could provide suggestions for ex-ante evaluations, which would be significant to strengthen national emergency preparedness capacity [59]. In emergency management information system, the GIS package should be emphasized to integrate and analysis multi-source data timely.

In the further post-earthquake recovery, the quality of construction should be emphasized. Especially in urban areas, where most residential buildings are made from concrete and brick, the main danger comes from collapsing buildings and falling debris. This threat is increased by the fact that many buildings in Chinese cities are high-rise. However, since the 1990s codes for construction and planning have been specified, which has led to higher levels of earthquake-resistance and better construction standards. The danger posed by earthquakes can be limited by building structures that can withstand earthquakes and will retain sufficient structural integrity to avoid collapse [60].

Disease surveillance systems should be strengthened, which is generally sufficient for controlling transmission of epidemic diseases. Usual post-disaster sanitation measures should be taken, especially the cardiovascular disease prevention [61] and mental health awareness [62]. Early implementation of immunization campaigns probably has a protective effect, and vaccination is recommended each time non-immunized populations are moved to camps.

All natural disasters are unique in that the affected regions have different social, economic, and health backgrounds, but many similarities exist, and knowledge about these can ensure that the health and emergency medical relief and limited resources are well-managed. Good disaster management must link data collection and analysis to the decision-making process [63]. How to use this rapid assessment in emergency management needs further discussion.

## 6. Conclusions

High resolution earth observation technology can provide a scientific basis for public health emergency management in the field of major disasters in the health and epidemic prevention. In the framework of rapid public health needs assessment: (1) casualties and injuries, (2) damaged medical facilities, (3) drinking water needs, (4) areas at risk of disease, and (5) temporary settlement sites were estimated after an earthquake using GIS technology and high-resolution remote sensing images in several hours, which is essential to improve the emergency response and primary healthcare delivery. Furthermore, this high-resolution image solution in disaster emergency monitoring and evaluation would be the demonstration of Chinese satellites application in public health. This article will be of great significance in helping policy makers effectively improve health service ability and public health emergency managers in prevention and control of infectious diseases and risk assessment.

## Figures and Tables

**Table 1:** The damage matrix of Type C and D structures (%).

**The Damage Matrix of Type C Structures (%)**					
**MMI**	**Undamaged**	**Slight**	**Middle**	**Severe**	**Destroyed**
VI	49	27.15	15.05	6.76	1.82
VII	28	21.29	22.07	20.27	8.36
VIII	12	16.33	23.09	30.22	18.28
IX	8	10.53	17.66	26.08	37.67
X	2.2	4.81	11.91	17.21	63.84
**The Damage Matrix of Type D Structures (%)**					
**MMI**	**Undamaged**	**Slight**	**Middle**	**Severe**	**Destroyed**
VI	32.5	26.5	22.5	16.5	2.5
VII	16.5	18.5	20	26	19
VIII	7	12	16.5	27	37.5
IX	2.5	8.5	14	25	50
X	0	1.5	7.5	17.5	73.5

**Table 2:** The correction factor for population density $f_{p}$.

Population Denstiy (Person/km^2^)	Less than 50	50~200	200~500	More than 500
$f_{p}$	0.8	1.0	1.1	1.2

**Table 3:** The time correction factor for an earthquake occurs at night.

MMI	VI	VII	VIII	IX	X
$f_{t}$	17	8	4	2	1.5

**Table 4:** Indicators of rescue point selection.

Principle	Indicator	Calculation
Nearest principle	Distance to the damaged residential area	Within 50–500 m
Safety Principle	Slope	Less than 15°
	Distance to river	100 m away
	Distance to landslide area	200 m away
Convenient transportation	Distance to road	Within 200 m
Size limitation	Minimum Size	2500 m^2^

**Table 5:** Assessment of casualties, injuries, medical resources, and water supplies after the Wenchuan earthquake.

County	2008 Population	2000 Gridded Population	Estimated Casualties	Estimated Injuries	Major Damage Area (Percentage)	Minor Damage Area (Percentage)	Medical Institutions (Number)	Damaged Medical Institutions (Number)	Water Supply Needs (L)
Anxian	476,072	497,308	12,275	36,825	44.2	55.8	24	15	5,239,637
Beichuan	157,341	159,098	9615	28,845	100	0	22	22	2,386,470
Dujiangyan	611,430	583,556	14,285	42,855	63.8	36.2	42	31	7,063,362
Maoxian	105,909	102,098	4399	13,197	100	0	25	25	1,531,470
Mianzhu	501,794	511,245	13,769	41,307	54	46	40	27	5,787,293
Pengzhou	754,925	773,772	9616	28,848	53.4	46.6	34	23	8,721,958
Pingwu	185,666	188,041	8557	25,671	0	0	27	27	2,820,615
Qingchuan	251,417	250,284	11,682	35,046	100	0	39	39	3,754,260
Shifang	420,225	400,650	6151	18,453	57.8	42.2	26	18	4,657,156
Wenchuan	114,138	109,523	5797	17,391	100	0	15	15	1,642,845
**Total**	3,578,917	3,575,575	96,146	288,438			294	242	43,605,066

**Table 6:** Risk assessment of infectious diseases.

Diseases	Risk ☆	Diseases	Risk ☆
Bacillary dysentery	++++	Visceral Leishmaniasis	++
Other infectious diarrhea	++++	Hemorrhagic fever with renal syndrome	++
Cholera	+++	Tetanus	++
Hepatitis A	+++	Malaria	+~++
Typhoid and paratyphoid	+++	Plague	+
Tuberculosis	+++	Hepatitis B	+
Acute upper respiratory tract infections	+++	Japanese Encephalitis	+
Rubella	+++	Leptospirosis	+
Mumps	+++	Dengue	+
Acute hemorrhagic conjunctivitis	+++	Rabies	+
Chicken Pox	+++	Schistosomiasis	+
Measles	++	Streptococcus suis	+
Meningococcal meningitis	++	Avian influenza/H5N1	+
Hand-foot-and-mouth disease	++	Syphilis/gonorrhea	+
Anthrax	++	SARS (Severe Acute Respiratory Syndromes)	-~+

☆ Note “-”, Very low or No Risk; “+”, Low Risk; “++”, Middle Risk; “+++”, High Risk; “++++”, Highest Risk; “~”, between.

## References

[1] Zhang L., Liu X., Li Y., Liu Z., Lin J., Shen J., Tang X., Zhang Y., Liang W. (2012). Emergency medical rescue efforts after a major earthquake: Lessons from the 2008 Wenchuan earthquake. Lancet.

[2] Liang Y. (2015). Satisfaction with economic and social rights and quality of life in a post-disaster zone in China: Evidence from earthquake-prone Sichuan. Disaster Med. Public Health Prep..

[3] Burchfiel B.C., Royden L.H., Van der Hilst R.D., Hager B.H. (2008). A geological and geophysical context for the Wenchuan earthquake of 12 May 2008, Sichuan, People’s Republic of China. GSA Today.

[4] Ni S., Wang W., Li L. (2010). The April 14th, 2010 Yushu earthquake, a devastating earthquake with foreshocks. Sci. China Earth Sci..

[5] Tang B., Zhang L. (2013). Ya’an earthquake. Lancet.

[6] Russo F., Rindone C., Trecozzi M.R. (2012). The role of training in evacuation. WIT Trans. Inf. Commun. Technol..

[7] Lillibridge S., Niji E., Burkle F. (1993). Disaster assessment: The emergency health evaluation of a population affected by a disaster. Ann. Emerg. Med..

[8] Ranjbar H.R., Ardalan A.A., Dehghani H., Saradjian M.R. (2017). Using high-resolution satellite imagery to provide a relief priority map after earthquake. Natl. Hazards.

[9] Feng T., Hong Z., Fu Q., Tong X. (2014). Application and prospect of a high-resolution remote sensing and geo-information system in estimating earthquake casualties. Natl. Hazards Earth Syst. Sci..

[10] Ranjbar H.R., Dehghani H., Ardalan A.R.A., Saradjian M.R. (2017). A GIS-based approach for earthquake loss estimation based on the immediate extraction of damaged buildings. Geomat. Natl. Hazards Risk.

[11] Kenny C. (2012). Disaster risk reduction in developing countries: Costs, benefits and institutions. Disasters.

[12] Rij E. (2016). An approach to the disaster profile of People’s Republic of China 1980–2013. Emerg. Disaster Rep..

[13] Zhang Y., Hao Y. (2008). Hazards and Strategies for Infectious Diseases Prevention and Control after Catastrophic Disaster of Earthquake. J. Sun Yat-Sen Univ. (Med. Sci.).

[14] Liu Y., Zhang Y., Liu X., Lin L., Liu Y., Peng Z. (2008). Disease surveillance and risk evaluation on the transmission of infectious disease in emergent status after earthquake. South China J. Prev. Med..

[15] Malilay J., Flanders W.D., Brogan D. (1996). A modified cluster sampling method for post-disaster rapid assessment of needs. Bull. World Health Organ..

[16] Akbari M.E., Farshad A.A., Asadi-Lari M. (2004). The devastation of Bam: An overview of health issues 1 month after the earthquake. Public Health.

[17] Ye F., Guo E., Liu J. (2013). Seismic damage investigation and analysis of water supply system in Lushan earthquake. World Earthq. Eng..

[18] Watson J.T., Michelle G., Maire A.C. (2007). Epidemics after natural disasters. Emerg. Infect. Dis..

[19] Chen Z., Chen X., Li Q., Chen J. (2013). The temporal hierarchy of shelters: A hierarchical location model for earthquake-shelter planning. Int. J. Geogr. Inf. Sci..

[20] Wagner R.M., Jones N.P., Smith G.S. (1994). Risk factors for casualty in earthquakes: The application of epidemiologic principles to structural engineering. Struct. Saf..

[21] Beck L.R., Lobitz B.M., Wood B.L. (2000). Remote sensing and human health: New sensors and new opportunities. Emerg. Infect. Dis..

[22] Hay S., Noor A., Nelson A., Tatem A. (2005). The accuracy of human population maps for public health application. Trop. Med. Int. Health.

[23] Aubrecht C., Freire S., Neuhold C., Curtis A., Steinnocher K. (2012). Introducing a temporal component in spatial vulnerability analysis. Disaster Adv..

[24] Freire S., Aubrecht C. (2012). Integrating population dynamics into mapping human exposure to seismic hazard. Nat. Hazards Earth Syst. Sci..

[25] Taskin G., Ersoy O.K., Kamasak M.E. (2015). Earthquake-induced damage classification from postearthquake satellite image using spectral and spatial features with support vector selection and adaptation. J. Appl. Remote Sens..

[26] Eguchi R.T., Mansouri B. (2005). Use of remote sensing technologies for building damage assessment after the 2003 Bam, Iran, earthquake—Preface to remote sensing papers. Earthq. Spectra.

[27] Wang F., Wang S., Zhou Y., Wang L., Yan F., Li W., Liu X. (2016). High Resolution Remote Sensing Monitoring and Assessment of Secondary Geological Disasters Triggered by the Lushan Earthquake. Spectrosc. Spectr. Anal..

[28] Cao C., Chang C., Xu M., Zhao J., Gao M., Zhang H., Guo J., Guo J., Dong L., He Q. (2010). Epidemic risk analysis after the Wenchuan Earthquake using remote sensing. Int. J. Remote Sens..

[29] Feng T., Hong Z., Wu H., Fu Q., Wang C., Jiang C., Tong X. (2013). Estimation of earthquake casualties using high-resolution remote sensing: A case study of Dujiangyan city in the May 2008 Wenchuan earthquake. Natl. Hazards.

[30] Huang T., Zhao T., Qu J., Wang S., Huang M., Wang S. (2008). The advantages of space information technology in serving Wenchuan earthquake relief decisions. Remote Sens. Technol. Appl..

[31] Hu S.Q. (2008). Research into “Information Lonely Island” in natural disasters with 5.12 Wenchuan earthquake as an example. J. Nanchang Coll..

[32] Brown D., Saito K., Liu M., Spence R., So E., Ramage M. (2012). The use of remotely sensed data and ground survey tools to assess damage and monitor early recovery following the 12.5. 2008 Wenchuan earthquake in China. Bull. Earthq. Eng..

[33] Zhang L.L., Liu Y., Liu X., Zhang Y. (2011). Rescue efforts management and characteristics of casualties of the Wenchuan earthquake in China. Emerg. Med. J..

[34] Zhong B., Chen L., Liu Y., Wu Z.S., Zhu H.Q., Lu D., Xu L., Zhang Y., Wang C.F., Xie M.K. (2013). Risk assessment of schistosomiasis transmission in earthquake-stricken areas after the Lushan Earthquake in Sichuan Province on April 20, 2013. Chin. J. Schistosomiasis Control.

[35] Yang X., Jiang D., Wang N., Liu H. (2002). Method of pixelizing population data. J. Geogr. Sci..

[36] Rabus B., Eineder M., Roth A., Bamler R. (2003). The shuttle radar topography mission—A new class of digital elevation models acquired by spaceborne radar. ISPRS J. Photogram. Remote Sens..

[37] Wei B., Nie G., Su G., Sun L., Bai X., Qi W. (2017). Risk assessment of people trapped in earthquake based on km grid: A case study of the 2014 Ludian earthquake, China. Geomat. Natl. Hazards Risk.

[38] Jaiswal K., Wald D., Earle P., Porter K.A., Hearne M. (2011). Earthquake casualty models within the USGS Prompt Assessment of Global Earthquakes for Response (PAGER) system. Human Casualties in Earthquakes.

[39] Yin Z. (1995). Earthquake Hazard and Loss Prediction.

[40] Ma Y., Xie L. (2000). Methodologies for assessment of earthquake casualty. Earthq. Eng. Eng. Vib..

[41] Fan S., Hu Y., Liu Z. (2015). Research of information extraction of city building based on a new object-oriented method. J. South China Norm. Univ..

[42] Greaney P., Pfiffner S., David W.D. (2011). Humanitarian Charter and Minimum Standards in Humanitarian Response.

[43] Zhang B., Wang F., Zhao Y., Geng H. (2008). A probe into the transitional allocation plan after the earthquake: A case study on the allocation plan of Daguan town. New Archit..

[44] Song X. (2009). Selections of locations, environmental protections and fire research for settlements of victims in the Wenchuan earthquake. Fire Sci. Technol..

[45] Chinese Center for Disease Control and Prevention (2008). Preliminary Risk Assessment of Major Infectious Diseases in Disaster Area after Sichuan Earthquake. http://www.chinacdc.cn/n272442/n272530/n273736/n342415/n3866878/n3867602/appendix/Preliminary%20Report%20on%20Communicable%20Diseases%20Risk%20Assessment%20and%20Interventions%20after%20Sichuan%20Earthquake_China%20CDC.doc.

[46] Kouadio I.K., Aljunid S., Kamigaki T., Hammad K., Oshitani H. (2012). Infectious diseases following natural disasters: Prevention and control measures. Expert Rev. Anti-Infect. Ther..

[47] Petal M. (2011). Earthquake casualties research and public education. Human Casualties in Earthquakes.

[48] Chen Z., Nie Z., Ma X., Wang J., Chen Z., Liu C., Zhong Y., Sun Y., Zhang H., Li J. (2008). Water quality monitoring and safety evaluation of Yingxiu town, the epicenter of the “5.12” Wenchuan earthquake. Bull. Acad. Mil. Med. Sci..

[49] Lemonick D.M. (2011). Epidemics after natural disasters. Am. J. Clin. Med..

[50] World Health Organization (2005). Acute water diarrhea outbreak. Weekly Morbidity and Mortality Report.

[51] World Health Organization (2006). Acute jaundice syndrome. Weekly Morbidity and Mortality Report.

[52] Schneider E., Hajjeh R.A., Spiegel R.A., Jibson R.W., Harp E.L., Marshall G.A., Gunn R.A., McNeil M.M., Pinner R.W., Baron R.C. (1997). A coccidiomycosis outbreak following the Northridge, Calif, earthquake. JAMA.

[53] Logue J.N. (1996). Disasters, the environment, and public health: Improving our response. Am. J. Public Health.

[54] Nathalie F., Viel J.F., Mauny F.J., Hoen B., Piarroux R. (2006). Negligible risk for epidemics after geophysical disasters. Emerg. Infect. Dis..

[55] Ting L., Wang F., Wen J., Ping Y. (2011). Rapid assessment of health needs after disasters: A systematic review. Chin. J. Emerg. Med..

[56] Jin Q., Chen K.H., Huang Y. (2008). How to carry out health and disease prevention work in Aba after Wenchuan earthquake. Chin. Evid. Based Med..

[57] Zhao L., Rodriguez-Llanes J.M., Wu Q., van den Oever B., Westman L., Albela M., Pan L., Chen G., Zhang D., Hughes M. (2012). Multiple injuries after earthquakes: A retrospective analysis on 1871 injured patients from the 2008 Wenchuan earthquake. Crit. Care.

[58] Peng H. (2011). China’s Health Challenges after the Yushu Earthquake. Prehosp. Disaster Med..

[59] Liu T. (2010). The significance of emergency preparedness highlighted by Yushu earthquake once again. J. Saf. Sci. Technol..

[60] Ye S., Zhai G., Hu J. (2011). Damages and Lessons from the Wenchuan Earthquake in China. Hum. Ecol. Risk Assess. Int. J..

[61] Teng A.M., Blakely T., Ivory V., Kingham S., Cameron V. (2017). Living in areas with different levels of earthquake damage and association with risk of cardiovascular disease: A cohort-linkage study. Lancet Planet. Health.

[62] Liu M., Wang L., Shi Z., Zhang Z., Zhang K., Shen J. (2011). Mental health problems among children one-year after Sichuan earthquake in China: A follow-up study. PLoS ONE.

[63] Giovanna C., Giuseppe M., Antonio P., Corrado R., Francesco R., Antonino V. (2016). Transport models and intelligent transportation system to support urban evacuation planning process. IET Intell. Transp. Syst..

